# Role of peroxisome proliferator-activated receptor gamma coactivator 1-alpha (PGC-1α) in denervation-induced atrophy in aged muscle: facts and hypotheses

**DOI:** 10.1186/2046-2395-2-13

**Published:** 2013-08-01

**Authors:** Gilles Gouspillou, Martin Picard, Richard Godin, Yan Burelle, Russell T Hepple

**Affiliations:** 1 Department of Critical Care, McGill University Health Centre, 687 Pine Ave West, Montreal, QC H3A 1A1, Canada; 2 Department of Medicine, McGill University, Royal Victoria Hospital, Montreal, QC, Canada; 3 Department of Kinesiology and Physical Education, McGill University, 475 Pine Ave West, Montreal, QC H2W 1S4, Canada; 4 Faculty of Pharmacy, Université de Montréal, 2940, Chemin de la polytechnique, Montreal, QC H3C 3J7, Canada; 5 Université du Québec À Montréal (UQÀM), Faculté des sciences Pavillon des sciences biologiques (SB), Local: SB-4640 141, Avenue du Président Kennedy, Montréal, Québec H2X 1Y4, Canada; 6 Center for Mitochondrial and Epigenomic Medicine − CMEM, Children's Hospital of Philadelphia, University of Pennsylvania, Colket Translational Research Center, 3501 Civic Center Blvd, Room 6100, Philadelphia, PA 19104, USA

**Keywords:** Skeletal muscle, Aging, Sarcopenia, PGC-1α, Denervation, Reinnervation

## Abstract

Aging-related loss of muscle mass, a biological process named sarcopenia, contributes to mobility impairment, falls, and physical frailty, resulting in an impaired quality of life in older people. In view of the aging of our society, understanding the underlying mechanisms of sarcopenia is a major health-care imperative. Evidence obtained from human and rodent studies demonstrates that skeletal muscle denervation/reinnervation cycles occur with aging, and that progressive failure of myofiber reinnervation is a major cause of the accelerating phase of sarcopenia in advanced age. However, the mechanisms responsible for the loss of myofiber innervation with aging remain unknown. The two major strategies that counteract sarcopenia, that is, caloric restriction and endurance training, are well known to protect neuromuscular junction (NMJ) integrity, albeit through undefined mechanisms. Interestingly, both of these interventions better preserve PGC-1α expression with aging, a transcriptional coactivator which has recently been shown to regulate key proteins involved in maintaining NMJ integrity. We therefore propose that the aging-related decline in PGC-1α may be a central mechanism promoting instability of the NMJ and consequently, aging-related alterations of myofiber innervation in sarcopenia. Similarly, the promotion of PGC-1α expression by both caloric restriction and exercise training may be fundamental to their protective benefits for aging muscle by better preserving NMJ integrity.

## Review

### Introduction

One of the most significant changes associated with normal aging is a progressive loss of muscle mass and strength, a biological process defined as sarcopenia [1]. Indeed, sarcopenia is considered as the major factor leading to mobility impairment, falls, and physical frailty in older people [2,3], dramatically impairing quality of life of afflicted individuals. Underscoring the magnitude of sarcopenia’s impact, in 2002, a study conducted in the United States revealed that approximately 64% and 31% of men and women aged 60 and older, respectively, had a height-normalized muscle mass that was below the lower boundary of the range seen in a healthy young adult (YA) population [4]. Further to this point, the health-care costs attributable to sarcopenia are considerable, where it was estimated at $18.5 billion dollars for the year of 2002 in the United States [4]. Since the proportion of individuals over 60 years old is rising steadily and is expected to reach 22% of the worldwide population in 2050 [5], unraveling the mechanisms involved in sarcopenia to promote development of the most effective therapeutic interventions is one of the major challenges facing health research.

The etiology of sarcopenia is extremely complex and continues to be the focus of intensive research. Many different biological mechanisms are proposed to contribute to sarcopenia, including a decrease in circulating levels of anabolic hormones and decrease in the anabolic response of aged myofibers [6,7], low-grade chronic inflammation [8-10], activation of proteolytic pathways and decreased protein synthesis [11-13], decline in satellite cell activation and proliferation [14-16], increased oxidative damage secondary to mitochondrial reactive oxygen species (ROS) overproduction, accumulation of mitochondrial dysfunction and activation of mitochondrial-mediated apoptosis [17], and alterations in the nervous system [18-20]. The relative contributions of all of these suspects are still unclear and require further study.

Compelling evidence collected over the last few decades places denervation, a potent inducer of muscle atrophy [21], as one of the most significant factors driving sarcopenia, particularly the selective atrophy of an increasing abundance of muscle fibers in advanced stages of sarcopenia where the functional consequences are most likely to have clinical impact [22]. As will be discussed in detail below, skeletal muscle fibers undergo progressive cycles of denervation and reinnervation with aging. In this scenario, muscle atrophy is thought to accelerate when the rate of denervation outpaces the rate of reinnervation. Support for this view is derived from the fact that two of the most efficient strategies to slow down sarcopenia, that is, endurance training (ET) and caloric restriction (CR), both positively influence the integrity of the neuromuscular junction (NMJ) in aged muscles [23]. However, the mechanism(s) driving the denervation/reinnervation cycles occurring with aging, as well as progressive failure in reinnervation, remain largely unknown.

In the present perspective paper, we will first briefly review the literature in support of denervation as a primary cause of aging-related muscle atrophy. We will then discuss the role that aging-related changes in peroxisome proliferator-activated receptor gamma coactivator 1-alpha (PGC-1α) expression may have in sparking denervation/reinnervation cycles and in turn, in precipitating aging muscle atrophy. To support our hypothesis, recent findings indicating that PGC-1α regulates the expression of key proteins involved in the maintenance of the NMJ, as well as the documented effects of CR and ET on muscle aging and PGC-1α, will be discussed.

### Denervation as a primary cause of sarcopenia

Compelling evidence exists that denervation is a central process in sarcopenia and experimental data supporting this view shows involvement from the peripheral nervous system all the way to myofibers. Details of this issue are discussed below.

At the spinal cord level, aging is associated with a gradual decrease in the motor neuron number. One of the very first studies that investigated the effect of aging on motor neuron number in humans was conducted in the late 1970s by Tomlinson and Irving. By estimating the number of motor neurons in the lumbosacral segments from cadavers of previously healthy individuals, these investigators reported that there was an average loss of total motor neurons throughout life of approximately 25%, with a sizeable fraction of subjects older than 60 years showing motor neuron counts of only 50% of those in early adult or middle age [24]. Interestingly, no sign of motor neuron loss was found up to the age of 60 years. These findings have been corroborated by many other studies that have also reported an aging-related reduction in motor unit (MU) number and/or a reduction in both the number and diameters of motor axons in human ventral roots [25-28]. Importantly, this aging-related decline in motor neuron number in humans is also a well-established feature of neuromuscular aging in rodents [22,23,29,30].

In line with these changes in the spinal cord, the number of downstream excitable MUs - with an MU defined as all of the muscle fibers that are innervated by a single motor neuron - was found to be decreased in aged individuals using the electrophysiological technique of motor unit number estimation (MUNE) [31-35]. In addition, several studies also reported that aging is associated with enlargement of MUs in humans [34-36] as well as in rats [37,38]. This lower number and larger size of MUs in aged muscles indicate that at least some of the myofibers that become denervated are reinnervated by axonal sprouting and expansion of existing MUs [39]. Furthermore, the characteristic fiber type grouping seen with increasing age in both humans [18,40-44] and animal models [45-47] is further evidence of repeating cycles of myofiber denervation followed by reinnervation through axonal sprouting from adjacent motor axons.

In addition to alterations in MU number and size, there are also marked changes at the level of the NMJ in both aging rodents [23,48-53] and humans [54,55]. Among the most convincing evidence that NMJs undergo aging-related deterioration was provided in a study conducted by Balice-Gordon in the late 1990s [53], in which an *in vivo* imaging approach was used, allowing the monitoring of the same NMJ repeatedly over time in living animals. Using these elegant techniques, Balice-Gordon observed that a significant portion of mouse NMJs display a gradual loss of motor terminal branches and dispersal of postjunctional acetylcholine receptor (AChR) clusters on myofibers with aging [53], such that the vast majority have undergone significant losses of pre- and postsynaptic sites in very advanced age (24 to 36 months) [53]. Many studies supporting these initial findings have since been conducted. For example, using transgenic mice in which motor axons were indelibly labeled with fluorescent proteins and by labeling AChRs with fluorescently tagged α-bungarotoxin, Valdez *et al*. reported that at 24 months in mice, around 80% of the NMJs were fragmented and approximately 15% of the NMJs were denervated in the tibialis anterior muscle [23]. Interestingly, in a study of Fisher 344 rats that did not yet exhibit signs of aging muscle atrophy, Deschenes *et al*. observed significant remodeling of the NMJ morphology in both soleus and plantaris (PL) muscles, although changes seen in soleus muscle were more modest [48]. On the basis of these results, Deschenes and colleagues suggested that impairment in the integrity of the NMJ morphology precedes aging muscle atrophy, and consequently that alterations of myofiber innervation play a causal role in sarcopenia.

At the myofiber level, strong support in favor of denervation has accumulated over the last few decades. Indeed, in elegant glycogen depletion experiments, Ansved and colleagues demonstrated that fibers belonging to individual MUs had a much higher probability of being beside one another with increasing age [37]. In addition, aged muscles are characterized by high level of myosin heavy chain (MHC) coexpression (that is, fibers expressing more than one MHC isoform) [42,45,46,56,57], a phenomenon thought to arise from aging-related denervation [22,58]. Among the arguments in favor of this interpretation is the fact that experimental/surgical denervation induces a high level of MHC coexpression within myofibers [59,60]. In addition, we recently demonstrated that MHC coexpression and myofiber atrophy in aged rat muscle are directly linked to a molecular marker of denervation [22]. Specifically, we showed that myofibers that expressed the sodium channel isoform - voltage-sensitive sodium channel 1.5 (Nav_1.5_) - an isoform that is only seen in adult muscle following denervation [61,62] - were on average 35% smaller than the innervated fibers, whereas innervated fibers in aged muscle were only 7% smaller compared with YA muscle [22]. Most impressively, 90% of the severely atrophied fibers (that is. cross-sectional area ≤1000 μm^2^) were positive for Nav_1.5_, and more than 70% of the fibers that coexpressed fast and slow MHC together were positive for Nav_1.5_. Therefore, available evidence implicates denervation as the primary cause of both MHC coexpression and myofiber atrophy in severely atrophied aging skeletal muscle [22].

**Figure 1:**
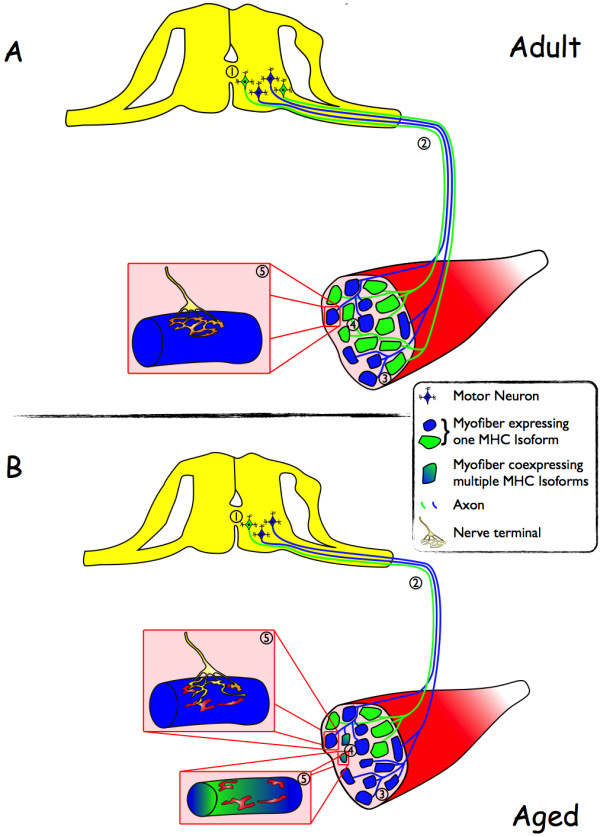
**Aging-related changes of the neuromuscular system: the central role of denervation.** To illustrate the main aging-related changes of the neuromuscular system that identify denervation as a primary cause of sarcopenia, schematic representations of adult **(A)** and aged **(B)** neuromuscular systems are presented. Key features of neuromuscular aging, all indicative of denervation, are highlighted as follow: loss of motor neurons (normally located in lamina IX of the spinal cord; not represented for clarity purposes) **(1)**, decrease in both axon number and diameter **(2)**, fiber type grouping **(3)**, increase in myosin heavy chain co-expression **(4)**, and appearance of fragmented or denervated neuromuscular junctions **(5)**. See main text for more details.

Taken together, the experimental findings discussed above (summarized in Figure 1) converge to indicate that denervation is a central process leading to sarcopenia, particularly so when the rate of muscle atrophy accelerates in very advanced age and is, therefore, most likely to precipitate functional impairment. The question that still remains is whether cycles of denervation and reinnervation are myofiber- or motor neuron-driven (or both). Although it may appear obvious to the reader that these alterations should arise from the progressive loss of motor neurons, strong rationale exists for the alternate possibility that the early changes in MU organization may arise from signals originating in the myofiber compartment. First, recent data shows that NMJ deterioration and histological markers of repeating denervation and reinnervation cycles in aging muscle occur prior to the loss of motor neurons in the spinal cord [63]. In addition, it has also been shown in mice selectively overexpressing the uncoupling protein 1 in skeletal muscle, an alteration which induced pathological uncoupling of mitochondrial oxygen consumption from adenosine triphosphate (ATP) production, that retrograde signals from the myofiber can lead to NMJ deterioration and motor neuron axonal die-back [64]. Further to these points, a mouse model engineered to overexpress neurotrypsin, an endogenous protease that cleaves and inactivates agrin at the NMJ, recapitulates key features seen in normally aging muscle including fiber type grouping, fiber loss, MHC coexpression and selective fiber atrophy, and this occurs without a decline in spinal cord motor neuron number [65]. In light of these findings, it therefore appears reasonable to think that changes at the myofiber level may be central components in initiating the aging-related denervation-reinnervation cycles. As will be discussed in the subsequent sections, recent findings converge to indicate that aging-related changes in PGC-1α expression in myofibers may be an important process triggering the onset and development of changes in the NMJ with aging.

### Protective effects of endurance training and caloric restriction in aging muscle: a role for PGC-1α

Among the most widely studied strategies to counteract sarcopenia are ET and CR. Both of these strategies are known to attenuate the age-related loss of muscle mass [66-68], as evidenced by higher muscle mass in old CR and ET animals as compared to old control animals [66,67] and higher fiber cross-sectional area in ET old humans as compared to old sedentary humans [68]. CR in particular not only attenuates aging muscle atrophy but also promotes a remarkable preservation of muscle contractile and metabolic capacities [66,69]. Interestingly, CR was also shown to attenuate the decline in muscle PGC-1α that occurs with normal aging. Although the initial interpretation of the consequences of maintenance of PGC-1α by CR was that this was preserving mitochondrial protein renewal and thus, mitochondrial function [69], more recent results suggest an alternate interpretation may apply. Specifically, although PGC-1α is best known for its role in promoting mitochondrial biogenesis, it also plays a key role in regulating the expression of several components of the NMJ in the muscle fiber [70]. As will be detailed below, this role of PGC-1α suggests the impact of both the decline in muscle PGC-1α with normal aging and its preservation by CR and ET may have significant impact on the integrity of the NMJ with aging.

**Figure 2:**
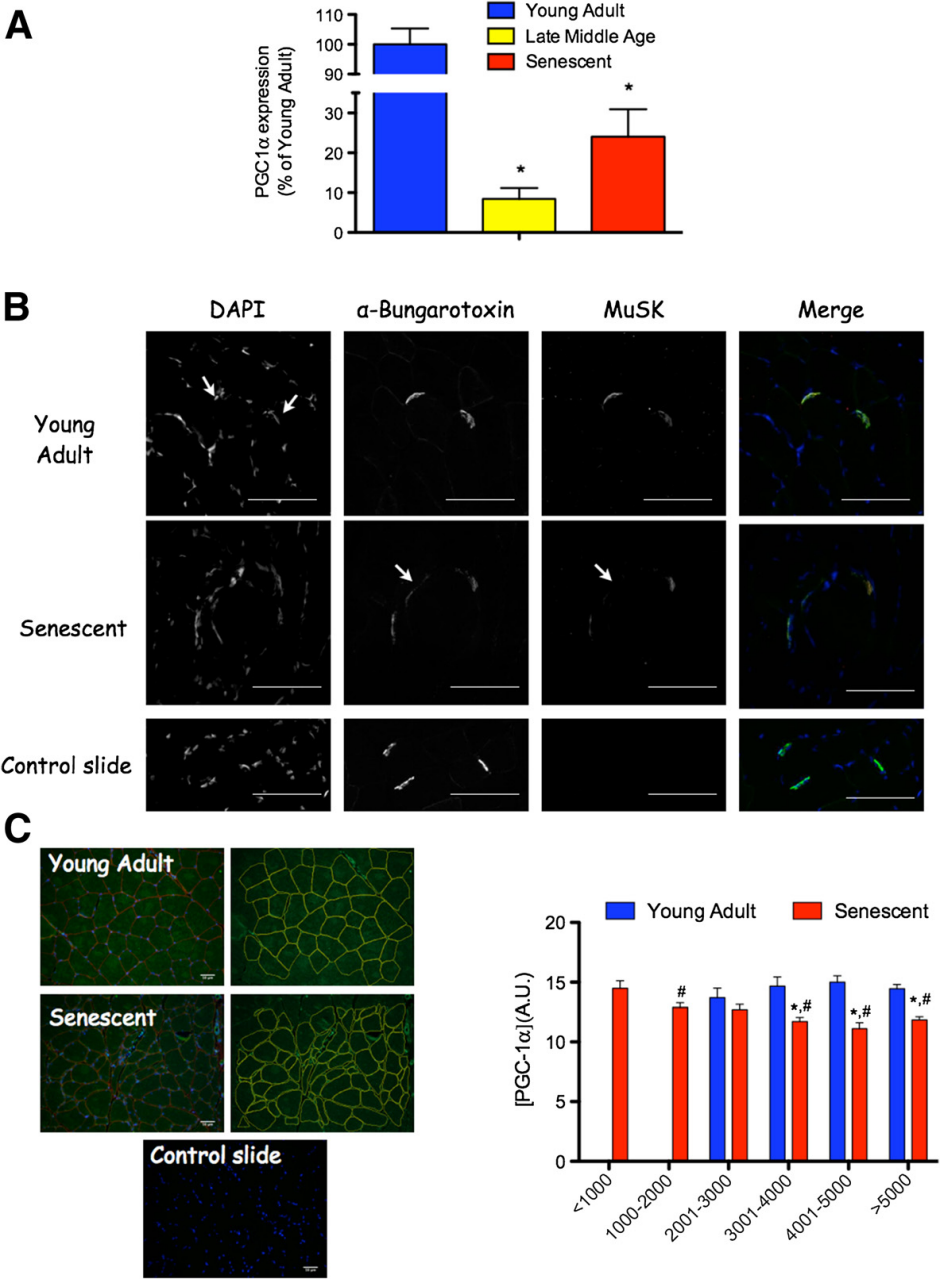
**Aging-related changes in PGC-1α and muscle-specific kinase (MuSK). (A)** Aging-related changes in PGC-1α expression. Data adapted from [69] (with permission from Oxford University Press), where PGC-1α expression was determined in gastrocnemius muscle of young adult (YA; 8 to 10 months) late middle-aged (LMA; 30 months) and senescent (SEN; 35 months) Fisher 344/Brown Norway F1 hybrids rats. Note the dramatic decrease in muscle PGC-1α expression from adulthood to LMA and its partial recovery from LMA to SEN. **(B)** Evidence for aging-related decrease in MuSK content at the neuromuscular junction (NMJ). MuSK protein content at the NMJ was determined *in situ* by immunolabeling plantaris cross-sections from YA (6 months) and SEN (35 months) rats with DAPI (labeling nuclei - blue in merge image), α-bungarotoxin (labeling acetylcholine receptors - green in merge image), and anti-MuSK antibody (kindly provided by Dr. Markus Rüegg; red in merge image) using protocols we previously described [22,57]. A control slide, for which the incubation with the anti-MuSK antibody was omitted, is presented at the bottom of panel B. White arrows point toward a NMJ having very low MuSK protein content in SEN muscle. **(C)** Aging-related changes in PGC-1α determined *in situ*. Cross-sections of the white (glycolytic) gastrocnemius region of one YA and SEN rat were immunolabeled for PGC-1α (green), dystrophin (red) and nuclei (blue) according to methods described in [22]. The anti-PGC-1α antibody was purchased from Millipore (AB3242; Millipore, Billerica, MA, USA). PGC-1α content was quantified by tracing each fiber using ImageJ (images on the right). A control slide, for which incubation with the anti-PGC-1α antibody was omitted, is presented at the bottom. The graph on the right presents PGC-1α content as a function of fiber size. *P <0.05 vs. YA, ^#^*P* <0.05 vs. small fibers (<1000 μm^2^). DAPI, 4′,6′-diamidino-2-phénylindole; PGC-1α, peroxisome proliferator-activated receptor gamma coactivator 1-alpha.

In an elegant study, Valdez *et al*. found in mice that were either calorie restricted or endurance trained there was superior maintenance of NMJ integrity during aging [23]. Although a remarkable diversity of mechanisms are regulated by ET and CR, one of the best characterized and common effects of both strategies is that they induce an increase in the expression of PGC-1α (see [71] for an extensive review).Therefore, on the basis of this fact and the known role of PGC-1α in regulating expression of key components of the NMJ, we speculate that CR- and ET-induced increase in PGC-1α mediates the protective effects of these interventions on the aging NMJ. Consistent with this view, transgenic mice with muscle-specific overexpression of PGC-1α exhibit an attenuated degradation of NMJ integrity at an age where muscle is beginning to show initial signs of aging [52]. These data therefore provide a strong rationale for the idea that PGC-1α is involved in aging-related alteration of NMJ stability. Additionally, both mRNA levels [52,69] and protein content of PGC-1α are known to be reduced in aging muscle [72,73] (Figure 2A), although this was not seen in a recent study of aging mice [74]. In summary, because of the protective influence of PGC-1α overexpression on NMJ integrity with aging [52], declines in muscle PGC-1α with aging could lead to impaired integrity of myofiber innervation, whereas upregulation of PGC-1α with CR and ET could explain the protection of the NMJ with aging by these interventions.

### PGC-1α in aging-related denervation

Given the fact that PGC-1α is usually considered as the master regulator of mitochondrial biology [75,76] it is possible that at least some of its protective effects on muscle innervation with aging [52] may be mediated by improved mitochondrial function. Indeed, alterations of different aspects of mitochondrial biology, including apoptotic signaling [17], altered energetics [77,78] and increased ROS production [79], are proposed to contribute to the development of sarcopenia. However, a recent comprehensive analysis of these aspects of mitochondrial function in four muscles experiencing different degrees of aging-related atrophy challenged this notion, as there was no relationship between degree of atrophy and mitochondrial dysfunction [80]. Indeed, the nature of the alterations in mitochondrial function in aging muscle, which included a modest elevation of ROS and sensitization of the mitochondria to an apoptotic challenge [80], were strikingly similar to the effects of surgical denervation on mitochondrial function [81]. Therefore, as these results suggest that intrinsic changes in mitochondrial function may not be a primary defect involved in aging-related atrophy, the prevention of muscle atrophy seen after ET, CR, or when PGC-1α is overexpressed, may be independent of PGC-1α’s effect on mitochondrial function. Further to this point, it is important to note that controversies about the role played by PGC-1α in mediating ET-induced mitochondrial biogenesis in skeletal muscle exist [82-84].

As mentioned above, recent findings indicate that PGC-1α is directly involved in regulating the expression of proteins required to maintain NMJ integrity. Using muscle-specific PGC-1α knockout and PGC-1α overexpressing mice, Handschin *et al*. recently demonstrated that PGC-1α regulates the expression of key proteins involved in the maintenance of the NMJ [70], including muscle-specific kinase (MuSK), a tyrosine kinase in muscle that coordinates signals involved in clustering of AChRs in the postsynaptic membrane of the NMJ [85]. The decrease in PGC-1α expression widely reported in aged muscles could thus trigger NMJ instability that ultimately leads to myofiber denervation through a decrease in MuSK expression and altered downstream signaling that is essential to maintaining the structural organization of the AChRs on the postjunctional membrane. In support of this hypothesis, we collected preliminary data suggesting that MuSK protein content at the level of the NMJ is decreased in the PL muscle of senescent (SEN) rats as compared to their YA counterparts (Figure 2B). Also consistent with an important role for a decline in MuSK signaling in causing aging muscle atrophy, a mouse transgenically modified to overexpress neurotrypsin [65], an endogenous protease that inactivates neural agrin [86] and thereby decreases MuSK activity [85], also causes precocious aging muscle atrophy with hallmark features we see in normally aging muscle (noted in the section “Denervation as a primary cause of sarcopenia”).

### How could PGC-1α drive aging-related reinnervation?

**Figure 3:**
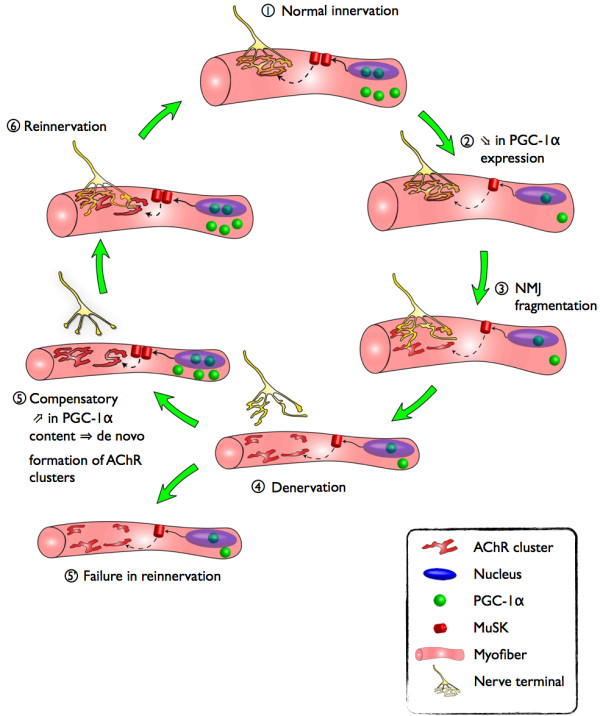
**PGC-1α in aging-related denervation/reinnervation cycles: a hypothetical mechanism.** In adult muscle **(1)**, PGC-1α is known to regulate expression of proteins involved in neuromuscular junction integrity, such as muscle-specific kinase (MuSK) and three acetylcholine receptor subunits. We hypothesize that decline in PGC-1α expression with aging (see Figure 2) leads to a decreased expression of MuSK and acetylcholine receptor subunits **(2)**, therefore promoting neuromuscular instability **(3)** and subsequent loss of innervation and decrease in fiber size **(4)**. We also hypothesize that changes in cellular conditions secondary to denervation (namely, an increase in mitochondrial reactive oxygen species generation) promotes an increase in PGC-1α expression which ultimately, through an increase in the expression of MuSK and acetylcholine receptor subunits **(5)**, promotes muscle fiber reinnervation and partial recovery of fiber size **(6)**. At advanced stages of aging, the blunted response of PGC-1α may prevent successful reinnervation and therefore aggravate the decrease in fiber size (5). PGC-1α, peroxisome proliferator-activated receptor gamma coactivator 1-alpha.

As mentioned previously, >90% of the very small fibers (<1000 μm^2^) whose accumulation tracks the accelerating trajectory of whole muscle atrophy with aging [45] appear to be denervated based upon expression of the denervation-specific sodium channel, Nav_1.5_[22]. Interestingly, these very small fibers contain high levels of protein carbonyls [87], indicating that these fibers undergo oxidative stress with aging. In explaining this observation, experimental denervation increases mitochondrial ROS production [88], and thus, it is reasonable to expect mitochondrial ROS generation to increase in the sporadically denervated myofibers seen in aging muscle. Interestingly, high levels of ROS can increase PGC-1α expression, either directly or indirectly through adenosine monophosphate-activated protein kinase (AMPK) activation [89]. Although AMPK hyperactivation can trigger the activation of a muscle atrophy program and has been suggested as a potential contributor to sarcopenia [90], we speculate that it might serve as an integrator of the increased mitochondrial ROS signal in denervated muscle fibers [88] to increase PGC-1α activity/expression. In turn, we hypothesize that this would facilitate an increase in MuSK that leads to reconstitution of the postsynaptic AChR cluster in preparation for reinnervation. In support of this hypothesis, we previously reported a partial recovery of PGC-1α expression in SEN rats as compared to their late middle-aged littermates [69] (see Figure 2A), an observation consistent with the high abundance of denervated myofibers at this age [22]. We also found, in a preliminary study, that in contrast to fibers with normal size (characterized by a significant decrease in PGC-1α content), atrophied fibers (<1000 μm^2^ in cross-sectional area) display a PGC-1α content similar to that seen in YA fibers (Figure 2C). Additionally, PGC-1α expression is upregulated in skeletal muscle of superoxide dismutase (SOD)^−/−^ mice, an animal model exhibiting both increased oxidative stress and severe signs of denervation [91,92]. Thus, an increase in PGC-1α expression following aging-related denervation, followed by an upregulation of MuSK (see above) and subsequent reaggregation of the postsynaptic AChRs, could explain the reinnervation phenomenon seen with muscle aging (Figure 3).

Finally, a blunted response of the AMPK/PGC-1α axis may undermine reinnervation in more advanced aged muscle. Indeed, the AMPK/PGC-1α axis has been shown to be less responsive in aged skeletal muscle [93]. In this context, it is interesting to consider that 5 or 7 months of ET in rats from middle age to senescence did not increase muscle PGC-1α content [94], suggesting that the response of PGC-1α to metabolic stimuli is blunted at advanced stages of aging. This blunted response of PGC-1α may impair transcriptional activation of NMJ components and lead to failure in reinnervation. This would explain why at advanced stages of aging denervation rates surpass reinnervation rates, leading to a marked accumulation of small angular denervated myofibers and causing a marked acceleration of muscle atrophy [22,45].

## Conclusions

As emphasized in the present review, data collected over the last few decades position denervation as a primary cause of sarcopenia. Here, we present a strong rationale for proposing that cyclic oscillations in PGC-1α play a key role in the denervation/reinnervation cycles seen in aged muscle, through the transcriptional regulation of key proteins involved in the maintenance of NMJ integrity (Figure 3). On this basis, we argue that further studies testing this hypothesis will provide a basis for more broadly understanding the therapeutic potential of PGC-1α as a countermeasure for sarcopenia [95], as well as further illuminating the mechanisms of NMJ deterioration in aging muscle. Of particular importance will be testing the efficacy of PGC-1α at ages where whole muscle atrophy becomes severe and thus most likely to yield clinical consequence.

## Abbreviations

AChRs: Acetylcholine receptors; AMPK: Adenosine monophosphate-activated protein kinase; ATP: Adenosine triphosphate; CR: Caloric restriction; DAPI: 4*′*,6*′*-diamidino-2-phénylindole; ET: Endurance training; MHC: Myosin heavy chain; MU: Motor unit; MUNE: Motor unit number estimation; MuSK: Muscle-specific kinase; Nav1.5: Voltage-sensitive sodium channel 1.5; NMJ: Neuromuscular junction; PGC-1α: Peroxisome proliferator-activated receptor gamma coactivator 1-alpha; PL: Plantaris; ROS: Reactive oxygen species; SEN: Senescent; SOD: Superoxide dismutase; YA: Young adult.

## Competing interests

The authors declare that they have no competing interests.

## Authors’ contributions

GG and RG carried out the experiments and analyzed the data. GG and RTH wrote the manuscript. MP, RG and YB revised and edited the manuscript. All authors read and approved the final manuscript.
